# Study on the Rheological Properties of Aged SBS-Modified Asphalt Rejuvenated with Soybean Oil and Crumb Rubber

**DOI:** 10.3390/ma19204274

**Published:** 2026-10-09

**Authors:** Yang Chen, Chen Sun, Qinghao Ji, Yujuan Zhang

**Affiliations:** College of Civil Engineering and Transportation, Yangzhou University, Yangzhou 225127, China; 231404602@stu.yzu.edu.cn (Y.C.); mz120231084@stu.yzu.edu.cn (C.S.); mz120241106@stu.yzu.edu.cn (Q.J.)

**Keywords:** aged SBS-modified asphalt, soybean oil, crumb rubber, synergistic rejuvenation, rheological property, microscopic characterization

## Abstract

**Highlights:**

- CR compensates for the ASMA high-temperature performance loss caused by soybean oil rejuvenation.
- A continuous elastic network is constructed by CR to improve the rheological properties of ASMA.
- Beyond physical diffusion, chemical interactions occur during soybean oil rejuvenation of ASMA.
- Competitive adsorption of soybean oil exists between CR and residual SBS modifiers within ASMA.

**What is the implications of the main finding?**

- The soybean oil/CR synergistic system achieves balanced high- and low-temperature performance for ASMA.

**Abstract:**

To address the issue that a single soybean oil rejuvenator weakens the high-temperature performance of aged SBS-modified asphalt, soybean oil and crumb rubber (CR) were used to construct a synergistic composite rejuvenation system for long-term aged SBS-modified asphalt (ASMA). Conventional property tests, dynamic shear rheometer (DSR) tests, bending beam rheometer (BBR) tests, and microstructural characterization were performed to investigate the macroscopic rheological properties and microscopic mechanisms of rejuvenated asphalt. The results indicate that soybean oil can restore the low-temperature performance of ASMA, yet it weakens its high-temperature deformation resistance. Incorporating CR into the 5 wt% soybean oil rejuvenated system effectively compensates for this drawback. As the CR content increases, the high-temperature deformation resistance, low-temperature crack resistance, fatigue life, and thixotropy of the rejuvenated asphalt improve significantly. Specifically, as the CR content increases from 0 wt% to 25 wt%, the rutting factor at 64 °C increases 14.7-fold, the fatigue life at 5% strain increases 13.7-fold, the low-temperature creep stiffness at −24 °C decreases by 17.1%, and the creep rate increases by 51.8%. The balanced optimization of high- and low-temperature rheological performance of the rejuvenated asphalt can be interpreted from multi-scale characterization results. On the one hand, weak interfacial chemical reactions occur during soybean oil rejuvenation, while CR mainly exerts physical swelling and filling effects. On the other hand, the system gradually evolves from dispersed-phase filling to a continuous network as the CR content increases. In addition, the likely competitive adsorption of soybean oil between CR and residual SBS modifiers promotes the formation of the continuous phase. This study provides a feasible technical reference for the synergistic rejuvenation of aged SBS-modified asphalt using bio-oil and CR.

## 1. Introduction

SBS-modified asphalt (SMA) has been widely adopted for high-grade highways owing to its outstanding high- and low-temperature performance [1,2]. Nevertheless, under the combined action of long-term thermal oxygen aging and repeated traffic loading, a large number of early-built asphalt pavements have reached their design service life, generating hundreds of millions of tons of reclaimed asphalt pavement (RAP) every year [3]. Efficient recycling and resource utilization of RAP have accordingly become a critical issue demanding urgent solutions in pavement engineering. Asphalt aging is not only a major bottleneck limiting the high-content application of RAP but also a primary trigger for premature cracking in recycled pavements [4,5]. Against this background, high-performance rejuvenation technologies for aged SBS-modified asphalt (ASMA) have attracted substantial research attention worldwide [6].

Thermal oxidative aging dominates the aging process of asphalt pavements. During aging, the loss of light fractions contributes to overall hardening and embrittlement of asphalt, accompanied by marked degradation in pavement performance [7]. Conventional rejuvenation strategies generally restore asphalt performance by incorporating light-oil-based rejuvenators to replenish depleted light fractions [8]. In recent years, plant-oil-derived bio-rejuvenators have attracted growing research interest for aged asphalt rejuvenation owing to their environmental friendliness and abundant raw material sources. Existing studies have demonstrated that vegetable oils, including soybean oil, palm oil, sunflower oil, and castor oil, can effectively mitigate the hardening and embrittlement of aged asphalt and restore its service performance, providing new insights for high-content RAP recycling [9,10,11]. Among these candidates, soybean oil stands out as a promising plant-oil-based rejuvenator owing to its high output, low cost, and favorable compatibility with asphalt.

For base asphalt rejuvenation, Zhang et al. [12] verified via molecular dynamics simulations that soybean-oil-based rejuvenators can greatly increase the diffusion coefficients of saturates and aromatics within aged asphalt, facilitate the replenishment of light fractions, and contribute to the recovery of low-temperature performance. Rai et al. [13] revealed through macroscopic laboratory tests that waste soybean oil can effectively rejuvenate long-term aged asphalt and endow it with improved secondary-aging resistance and fatigue resistance. In terms of aged ASMA rejuvenation, Chen et al. [14] reported that waste soybean oil realizes physical rejuvenation by compensating for light fractions to counteract the accumulation of large molecular substances induced by aging, which remarkably improves the low-temperature performance of aged asphalt. Further research by Wen et al. [15] confirmed that soybean oil can significantly restore the fatigue life and self-healing capability of aged SBS-modified asphalt. Collectively, these previous findings validate that soybean-oil-based rejuvenators exhibit notable effectiveness in enhancing the low-temperature and fatigue performance of aged asphalt.

Although soybean-oil-based rejuvenators can notably improve the low-temperature and fatigue performance of aged asphalt, their sole application still has notable limitations. On the one hand, the strong softening effect of soybean oil tends to degrade the high-temperature rutting resistance of rejuvenated asphalt, and its optimal dosage generally ranges from 3 wt% to 6 wt% of asphalt binder [16]. On the other hand, neat soybean oil rejuvenators mainly function through physical dilution, which cannot fully compensate for the elasticity and high-temperature performance lost due to SBS degradation, resulting in asynchronous recovery between high- and low-temperature performance [17,18]. In general, although SBS-modified asphalt rejuvenated by waste vegetable oils can restore low-temperature and fatigue performance to the level of virgin asphalt, its elastic recovery and thermal stability still require further improvement [19,20]. Accordingly, the performance of aged SBS-modified asphalt (ASMA) cannot be fully restored by soybean oil alone.

To address the performance imbalance induced by single light-oil rejuvenators, researchers have explored multi-component composite rejuvenation strategies. Shi et al. [21] blended virgin SMA with ASMA and verified that the incorporation of SBS fractions could enhance the high-temperature rutting resistance and low-temperature performance of rejuvenated asphalt. Li et al. [22] achieved a favorable balance between high- and low-temperature performances of rejuvenated asphalt by optimizing the blending ratio of a light-oil rejuvenator and virgin SMA. Moreover, advances have also been achieved in the development of reactive rejuvenators. Zhang et al. [23] and Cao et al. [24] introduced isocyanate active functional groups to form chemical bridging effects, which improved the storage stability and high-temperature performance of rejuvenated systems. Chen et al. [25] and Shi et al. [26] validated the feasibility of “soybean oil + polymer” synergistic rejuvenation through laboratory composite rejuvenation experiments and molecular dynamics simulations, respectively. They reported that such functional systems could simultaneously restore asphalt binder fractions and provide supporting polymer networks. Nevertheless, the above-mentioned approaches are often limited by high costs or complex fabrication procedures.

Crumb rubber (CR), a polymeric modifier for asphalt, features abundant sources and low cost. Crumb-rubber-modified asphalt exhibits satisfactory high- and low-temperature performance and anti-aging properties [27,28]. On this basis, Zhang et al. [29] proposed a technical scheme for utilizing CR to synergistically rejuvenate ASMA upon light-oil rejuvenation. Their work demonstrated that CR served as a secondary modifier for rejuvenated ASMA and effectively improved its high-temperature performance. This technical concept aligns with the waste-to-waste green recycling philosophy reported by Mohamed et al. [30]. Furthermore, notable synergistic effects exist between soybean oil and CR. Soybean oil acts as a swelling agent for CR to facilitate its de-crosslinking and desulfurization degradation, improving the dispersion and compatibility of CR within the asphalt matrix and mitigating technical drawbacks such as excessive viscosity and poor storage stability of crumb-rubber-modified asphalt [31,32].

The rejuvenation performance of light-oil/polymer composite technology is predictable. However, when the soybean-oil-based rejuvenation scheme is compounded with other polymers, it still faces the critical limitation of high material cost despite achieving a desirable performance balance. In the system of synergistically rejuvenating aged asphalt via light oil and CR compounding, the multi-phase interfacial interaction mechanisms among different types of oil, CR, and aged asphalt exhibit notable heterogeneity, making it difficult to establish a unified performance rejuvenation principle. Currently, research on the regeneration system consisting of soybean oil and CR remains very limited. Meanwhile, in existing studies, whether CR is used for secondary modification rejuvenation of aged asphalt or directly incorporated into modified asphalt, the influence of CR on the low-temperature performance of the system is inconsistent across different working conditions, which fails to provide a reliable basis for the precise regulation of low-temperature crack resistance.

Soybean oil supplements the light components of asphalt, and CR constructs an elastic network. These two effects can produce a synergistic enhancement. Moreover, competitive adsorption of soybean oil among different phases may promote the formation of the co-continuous phase, thereby optimizing both the high- and low-temperature properties of ASMA. Therefore, based on this hypothesis, soybean oil and CR were used to synergistically rejuvenate ASMA in this work. Soybean oil was used to replenish the light fractions lost from the aged asphalt binder, while the elastic network structure of CR was used to compensate for performance deficiencies caused by SBS degradation to achieve synergistic rejuvenation of ASMA. Conventional physical property tests, dynamic shear rheometer (DSR) tests, and bending beam rheometer (BBR) tests were conducted to systematically explore the regulatory effects of this low-cost combination on the performance of ASMA and its internal rejuvenation mechanism. This research can provide new insights and theoretical foundations for the efficient and green rejuvenation of aged SBS-modified asphalt.

## 2. Materials and Methods

### 2.1. Materials

The SBS-modified asphalt (SMA) used in this study was supplied by Jiangsu Expressway New Materials Technology Co., Ltd. (Nantong, China), with an SBS modifier content of 4 wt%. The detailed technical characteristics of the SBS modifier are listed in Table S1 of the ESI. The crumb rubber (CR) used in this study was purchased from a mineral processing plant in Hebei Province, with a particle size of 60 mesh. The soybean oil used in this research was commercially available. The detailed performance parameters of the SMA, the short-term aged SBS-modified asphalt (S-SMA), and the long-term aged SBS-modified asphalt (ASMA) are listed in Table 1, and the performance parameters of CR can be found elsewhere [29]. The basic characteristics of these materials are listed in Tables S2 and S3 of the ESI.

### 2.2. Preparation Methods of the Rejuvenated Asphalts

The artificially aged SBS-modified (ASMA) asphalt was prepared by conducting a rolling thin film oven test (RTFOT) and a pressure aging vessel (PAV) test sequentially according to Chinese standards T 0610-2011 and T 0630-2025 of JTG 3410-2025, respectively. The first stage was the RTFOT conducted at 163 °C for 85 min to simulate short-term thermal oxidative aging during asphalt pavement construction. Subsequently, the PAV test was performed at 110 °C under 2.1 MPa for 20 h to further simulate the long-term field aging effect of asphalt after several years of service.

The rejuvenation in this study targets the long-term aged SBS-modified asphalt (ASMA). The ASMA was heated in an oven at 170 °C to reach a fluid state, and then soybean oil was mixed with the molten asphalt at a stirring speed of 300 rpm for 15 min to prepare the soybean-oil-rejuvenated SBS-modified asphalt (ASMA-S). The CR was subjected to a 90 min swelling treatment in the 5 wt% soybean-oil-rejuvenated ASMA at 170 °C, followed by a 60 min shearing process at approximately 170 °C using a high-speed shear mixer at 3000 rpm. The dosages of soybean oil and CR were determined based on the mass of the ASMA. The resulting rejuvenated ASMA containing soybean oil and CR is designated as ASMA-S-C-*x*, where *x* represents the CR content. The CR content ranges from 0 wt% to 25 wt%, in 5 wt% increments, covering the entire structural evolution from dispersed CR filling to a quasi-continuous CR network.

### 2.3. Characterizations

#### 2.3.1. Conventional Physical Properties

The conventional physical properties of the rejuvenated asphalt were tested in accordance with relevant standards [33], including penetration at 25 °C, softening point, ductility at 5 °C, and Brookfield viscosity at 135 °C. More than three tests were performed for each property, and the average values were obtained.

#### 2.3.2. Dynamic Shear Rheometer (DSR)

Rheological tests were performed using a DHR-2 rotational rheometer manufactured by TA Instruments (New Castle, DE, USA), equipped with a pair of 25 mm diameter parallel plates. The test gap was set to 1.0 mm, and the applied strain was controlled at 0.2%. Strain-sweep tests were conducted to confirm that all measurements were within the linear viscoelastic region (as shown in Figure S1 of the ESI). The test program included a temperature–frequency sweep test, a multiple stress creep recovery (MSCR) test, a linear amplitude sweep (LAS) fatigue test, and a thixotropy test to evaluate medium- and high-temperature properties.

The temperature–frequency sweep test was conducted in accordance with the specification T 0628-2011, with the temperature ranging from 52 °C to 82 °C and the scanning frequency varying from 0.1 Hz to 10 Hz.

The MSCR test was carried out at 64 °C, with all test parameters set following the protocol specified in T 0647-2025. The tests were performed following a two-stage constant stress loading protocol. Each creep–recovery cycle consisted of a 1 s constant stress loading phase followed by a 9 s zero-stress unloading recovery phase. A total of 30 consecutive cycles were implemented; 20 cycles were conducted at a low stress level of 0.1 kPa, followed by 10 cycles at a high stress level of 3.2 kPa.

The LAS fatigue test was completed at 25 °C in strict accordance with the specification T 0648-2025. The predicted fatigue life at 2.5% and 5% strain can be obtained by the integrated viscoelastic continuum damage (VECD) fatigue characterization.

The thixotropy test was also performed at 64 °C. First, the sample was subjected to pre-shear treatment, followed by an upward shear rate scanning stage in which the shear rate was linearly increased from 0.01 s^−1^ to 0.5 s^−1^ at an increment of 0.01 s^−1^. After that, a downward scanning stage was initiated, in which the shear rate was linearly decreased back to 0.01 s^−1^ at the same step, and the stress–shear rate curve was synchronously collected throughout the entire process.

#### 2.3.3. Bending Beam Rheometer (BBR)

The low-temperature creep performance test of the rejuvenated asphalt was carried out using a bending beam rheometer (BBR, TE-BBR-F, Cannon Instrument Company, State College, PA, USA). The test was performed in accordance with the specification T 0627-2011, and the test temperature was set to −24 °C.

#### 2.3.4. Attenuated Total Reflectance–Fourier Transform Infrared (ATR-FTIR) Spectroscopy

ATR-FTIR spectroscopy was performed to characterize the variations in chemical structure of the samples using a Cary 610/670 micro-infrared spectrometer manufactured by Varian, Palo Alto, CA, USA. The test was configured with a scanning wavenumber range of 4000 cm^−1^ to 500 cm^−1^, a spectral resolution of 4 cm^−1^, and 32 consecutive repeated scans for each specimen.

#### 2.3.5. Differential Scanning Calorimetry (DSC)

DSC 8500 (PerkinElmer Co., Ltd., Waltham, MA, USA) was employed to characterize the thermal behavior of the rejuvenated asphalt samples. The entire test was conducted under a continuous nitrogen purge atmosphere, with the procedure programmed to heat from −60 °C to 100 °C at a constant heating rate of 5 K/min.

#### 2.3.6. Morphological Characterization

The micromorphology of the rejuvenated asphalt was observed using an LSM700 3D laser confocal microscope (Carl Zeiss Co., Oberkochen, Baden-Württemberg, Germany). This instrument was equipped with a blue optical filter system that delivers excitation wavelengths spanning 390 nm to 490 nm, and all observations were performed under a fixed magnification of 200×. Prior to imaging, the heated liquid rejuvenated asphalt was poured into a custom square mold, then allowed to naturally cool to ambient temperature to obtain a specimen with a smooth, flat testing surface. The particle size analysis was conducted using Nano Measurer 1.2 software.

## 3. Results

### 3.1. The Conventional Properties of Rejuvenated Aged SBS-Modified Asphalt

**Figure 1:**
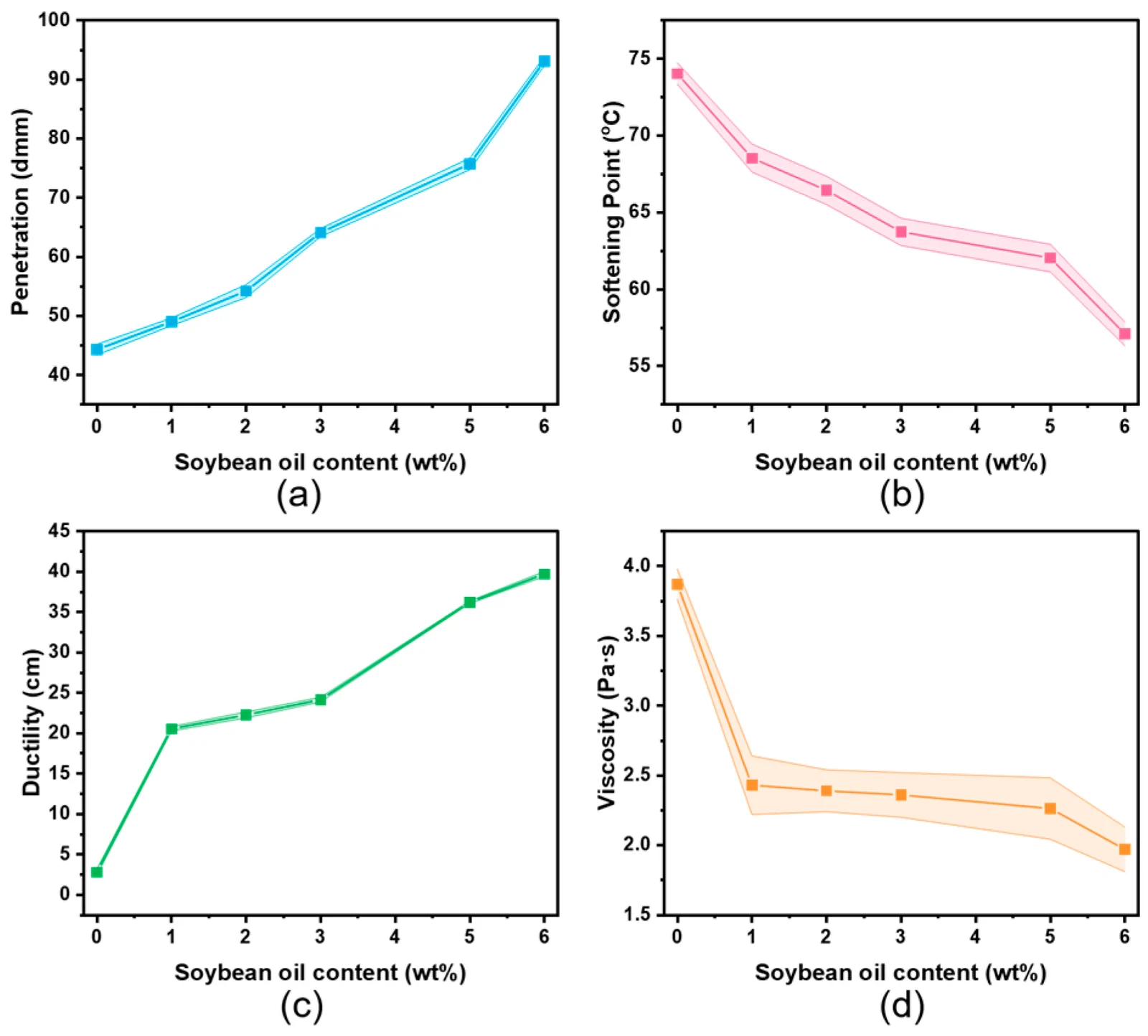
The (**a**) penetration at 25 °C, (**b**) softening point, (**c**) ductility at 5 °C and (**d**) Brookfield viscosity at 135 °C of ASMA-S.

The conventional properties of ASMA-S were evaluated at a dosage range of 0–6 wt%, and the results are presented in Figure 1. All performance indicators show monotonic variations with the increase in soybean oil content. The penetration (Figure 1a) and ductility (Figure 1c) increase monotonically, demonstrating that soybean oil replenishes the light fractions lost during asphalt aging, reduces the binder consistency, and effectively restores the low-temperature cracking resistance of aged asphalt. In Figure 1b,d, the softening point and viscosity decrease monotonically, indicating that the high-temperature property of the rejuvenated asphalt is slightly compromised, while the construction workability is significantly improved. The above performance evolution generally conforms to the typical softening mechanism of bio-based oil rejuvenators for aged asphalt. At 3 wt % and 4 wt % soybean oil dosages, the ductility recovery effect remains insufficient, with measured ductility values of 22.3 cm and 24.1 cm, respectively. Both values are far below the 31.0 cm value of unaged SBS-modified asphalt, indicating that the low-temperature ductility of aged ASMA cannot be fully restored. At the 5 wt.% dosage, the rejuvenated binder presents a ductility of 36.2 cm, which exceeds the ductility level of the original unaged SBS-modified asphalt, while maintaining a softening point of 62.0 °C. This value still retains sufficient high-temperature performance reserve, providing adequate space for the subsequent addition of CR to further construct the continuous elastic network and achieve the synergistic effect of high- and low-temperature rheological properties. When the dosage increases to 6 wt.%, the ductility can still increase marginally, but the softening point drops notably to 57.1 °C. Therefore, based on the comprehensive performance evaluation of the rejuvenated ASMA, a 5 wt% soybean oil dosage is determined as the baseline condition for subsequent incorporation of CR particles to construct a synergistic rejuvenation system targeting ASMA.

**Figure 2:**
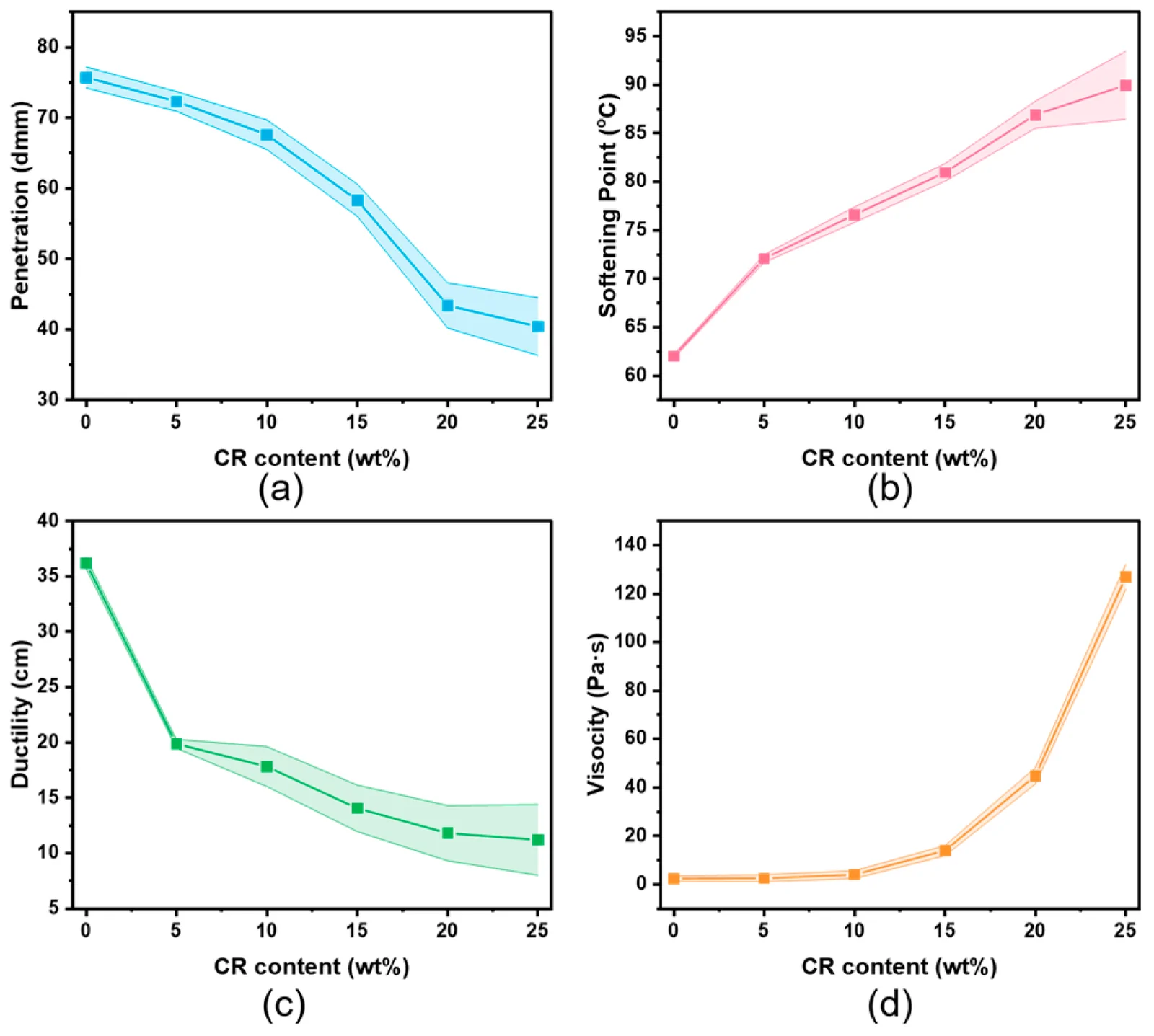
The (**a**) penetration at 25 °C, (**b**) softening point, (**c**) ductility at 5 °C and (**d**) Brookfield viscosity at 135 °C of ASMA-S-CR-*x*.

On the basis of ASMA rejuvenated by 5 wt% soybean oil, CR particles with contents ranging from 0 wt% to 25 wt% were further incorporated for synergistic rejuvenation, and the evolution of conventional properties is illustrated in Figure 2. The effect of CR content on performance is generally opposite to that of soybean oil content. The penetration (Figure 2a) and ductility (Figure 2c) decrease monotonically, while the softening point (Figure 2b) and viscosity (Figure 2d) increase monotonically. These results indicate that the incorporation of CR improves the consistency of the rejuvenated ASMA, compensates for the low consistency caused by soybean oil rejuvenation alone, restores and enhances the high-temperature deformation resistance of the rejuvenated ASMA, and offsets the weakening effect of single soybean oil rejuvenation on high-temperature performance. Nevertheless, this process reduces the low-temperature ductility of the rejuvenated ASMA to a certain extent, but the corresponding performance level is still superior to that of the unrejuvenated ASMA. Additionally, higher CR content will notably increase the flow resistance and reduce the construction workability. It is worth noting that the test error of each performance gradually increases with the rise in CR content, as reflected by the widened shaded error bands in the corresponding plots. This indicates that higher CR content tends to induce agglomeration, which may impair the dispersion uniformity of CR in the rejuvenated ASMA. In general, CR exerts a remarkably notable performance regulation effect on the soybean oil rejuvenation system. The synergistic effect of the two components compensates for the high-temperature performance deficiency of the bio-oil rejuvenator alone, achieving a balanced optimization of high- and low-temperature performances of the rejuvenated asphalt.

### 3.2. The High-Temperature Rheological Properties of Rejuvenated Aged SBS-Modified Asphalt

**Figure 3:**
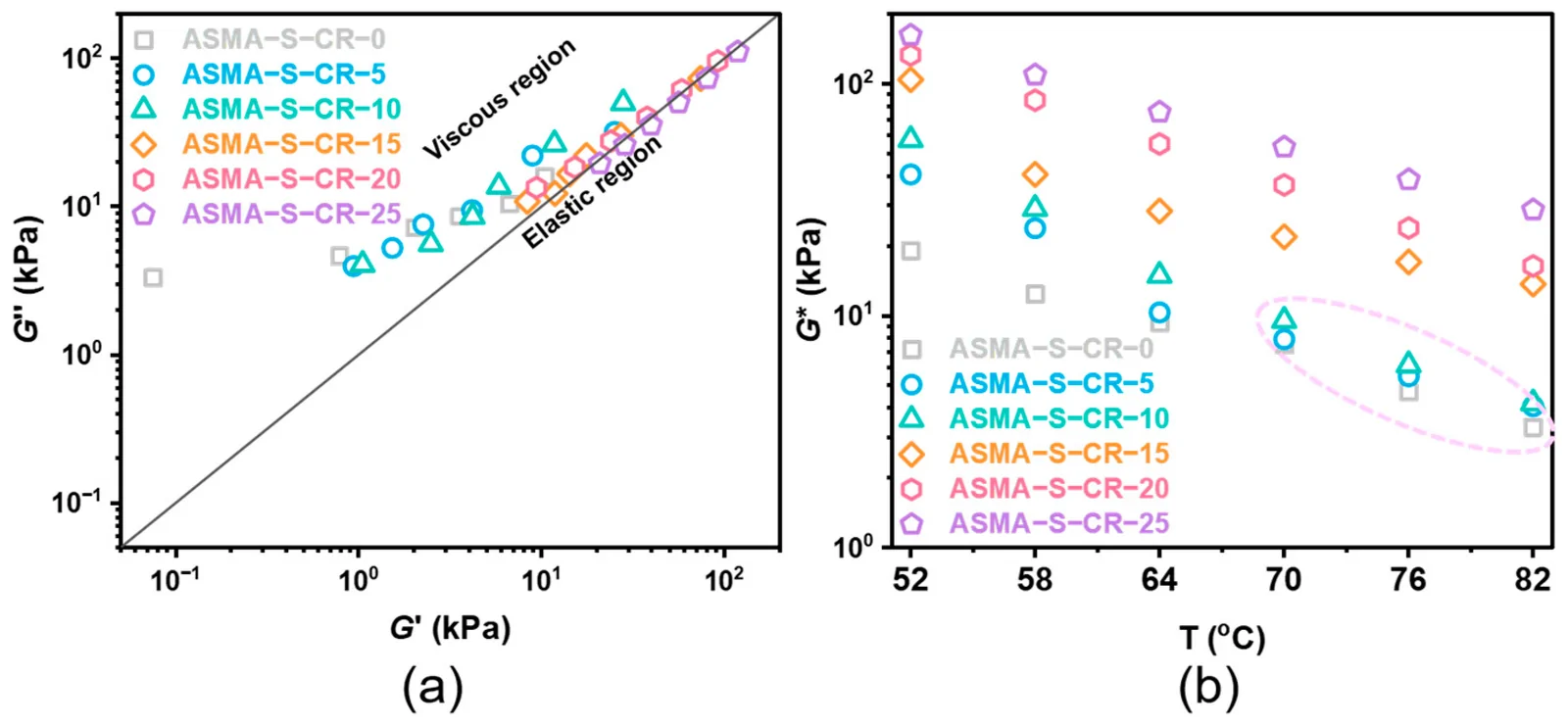
The (**a**) relationship between *G*′ and *G*″ and the (**b**) G* of ASMA-S-CR-*x*.

The relationship between storage modulus (*G*′) and loss modulus (*G*″) of different ASMA-S-CR-*x* samples is presented in Figure 3a, and these rheological curves can be used to characterize the elastic and viscous components in the rejuvenated asphalt. The black solid line in the figure clearly defines the demarcation threshold of viscoelastic behavior. The region above the line corresponds to the viscous-dominated rheological response, while the region below the line represents the elastic-dominated state. The test results clearly show that the rejuvenated SBS-modified asphalt without CR (ASMA-S-CR-0) exhibits typical viscous-dominated rheological properties [34]. Compared with ASMA-S-CR-0, the *G*′-*G*″ curves of the rejuvenated SBS-modified asphalt incorporated with CR show an overall shift towards the elastic-dominated region with the increasing content of CR, which indicates that the addition of CR can enhance the elastic response capacity of the rejuvenated asphalt system. Notably, when the CR content reaches 25 wt% (ASMA-S-CR-25), the rheological curve of the sample enters the elastic-dominated region. This phenomenon can be attributed to the physical volume filling effect of CR in the rejuvenated asphalt. Meanwhile, as an elastomer with a cross-linked network structure, CR can be swelled by the light components of asphalt, further strengthening the elastic contribution of the system. The above rheological results indicate that the incorporation of CR can regulate the rheological behavior of rejuvenated SBS-modified asphalt.

Figure 3b shows the complex shear modulus (G*) response characteristics of a series of rejuvenated SBS-modified asphalts with increasing temperature. As a key rheological index characterizing the shear deformation resistance and overall stiffness of asphalt, the variation in G* reflects the modification effect of CR. All CR-incorporated rejuvenated asphalts exhibit higher G* values than ASMA-S-CR-0, indicating that the introduction of CR improves the capability of rejuvenated asphalt to resist external shear failure. As test temperature increases, the G* of all samples shows a monotonic downward trend, indicating the typical temperature-sensitive property of this type of reclaimed modified asphalt. It is worth noting that the G* values of various samples show notable differences at low temperatures in the low CR content range, and these differences narrow as temperature increases (as marked by the circle). In contrast, for the rejuvenated system with high CR content, a distinct difference in G* remains among different samples even in the high-temperature test region. This phenomenon can be explained by the fact that for the system with low CR content, the rheological properties in the high-temperature region are still dominated by the asphalt matrix, and the modification effect of CR is limited. In contrast, for the system with high CR content, the cross-linked composite network formed by interconnected CR can sustain the modification effect over a wide temperature range, achieving long-term stiffness enhancement.

**Figure 4:**
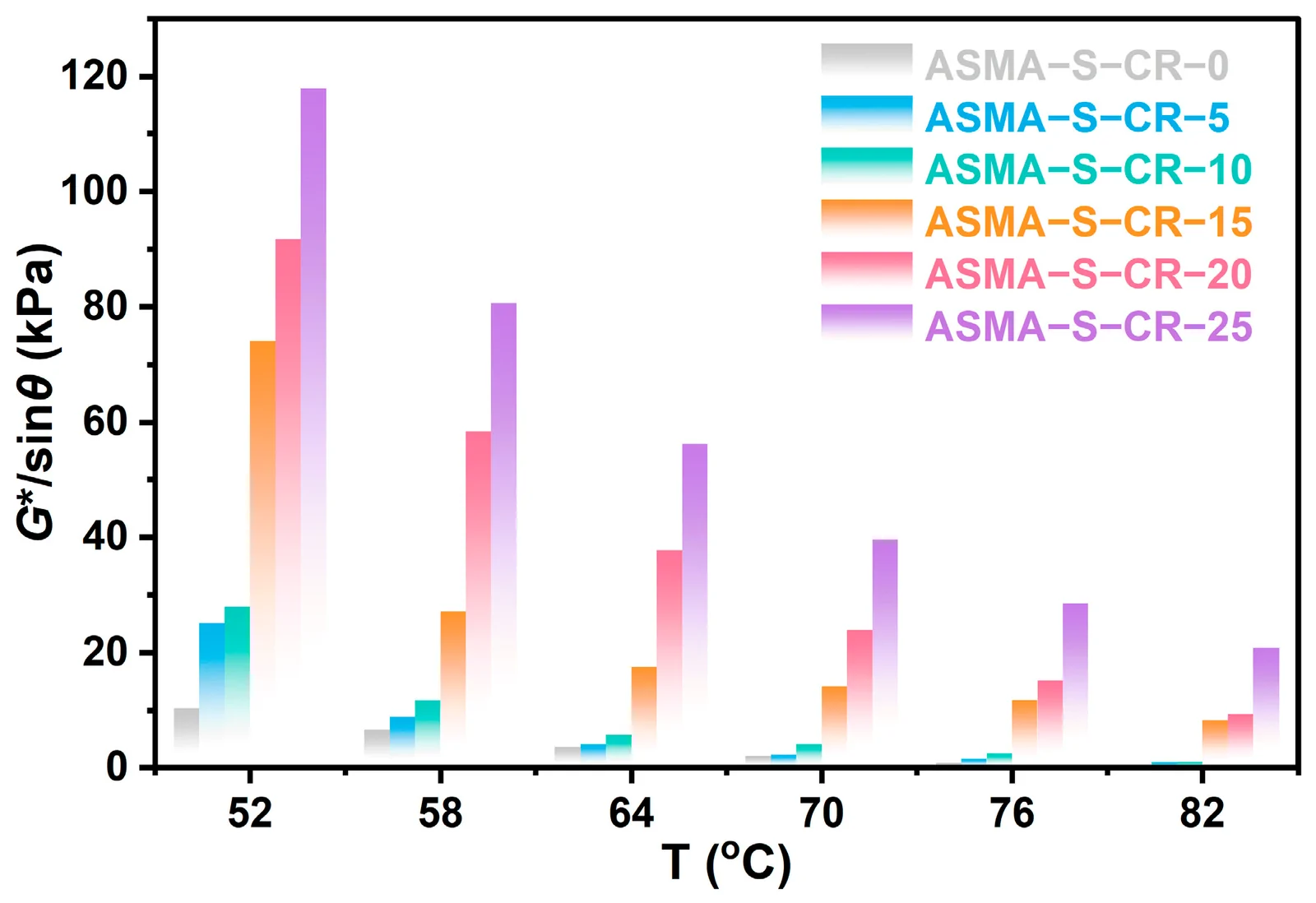
The G*/sin*δ* of ASMA-S-CR-*x*.

The rutting factor (G*/sin*δ*) calculated from the DSR test results is presented in Figure 4. All tested samples exhibit a monotonic decreasing trend of G*/sin*δ* as temperature rises. Meanwhile, the G*/sin*δ* of the rejuvenated asphalt increases with increasing CR content across the entire test temperature range. This result indicates that CR modification can improve the high-temperature rutting resistance of rejuvenated asphalt, consistent with the conclusions reported in existing literature [29,35]. Notably, in the high-temperature test region, the G*/sin*δ* of the rejuvenated asphalt with low CR content approached zero, which indicates that the system entered a viscous flow state and its rheological behavior is dominated by the asphalt. In contrast, the rejuvenated asphalt with relatively high CR content can still maintain considerable rutting resistance, and this retained performance advantage is mainly attributed to the stable elastic network structure formed by CR inside the asphalt.

**Figure 5:**
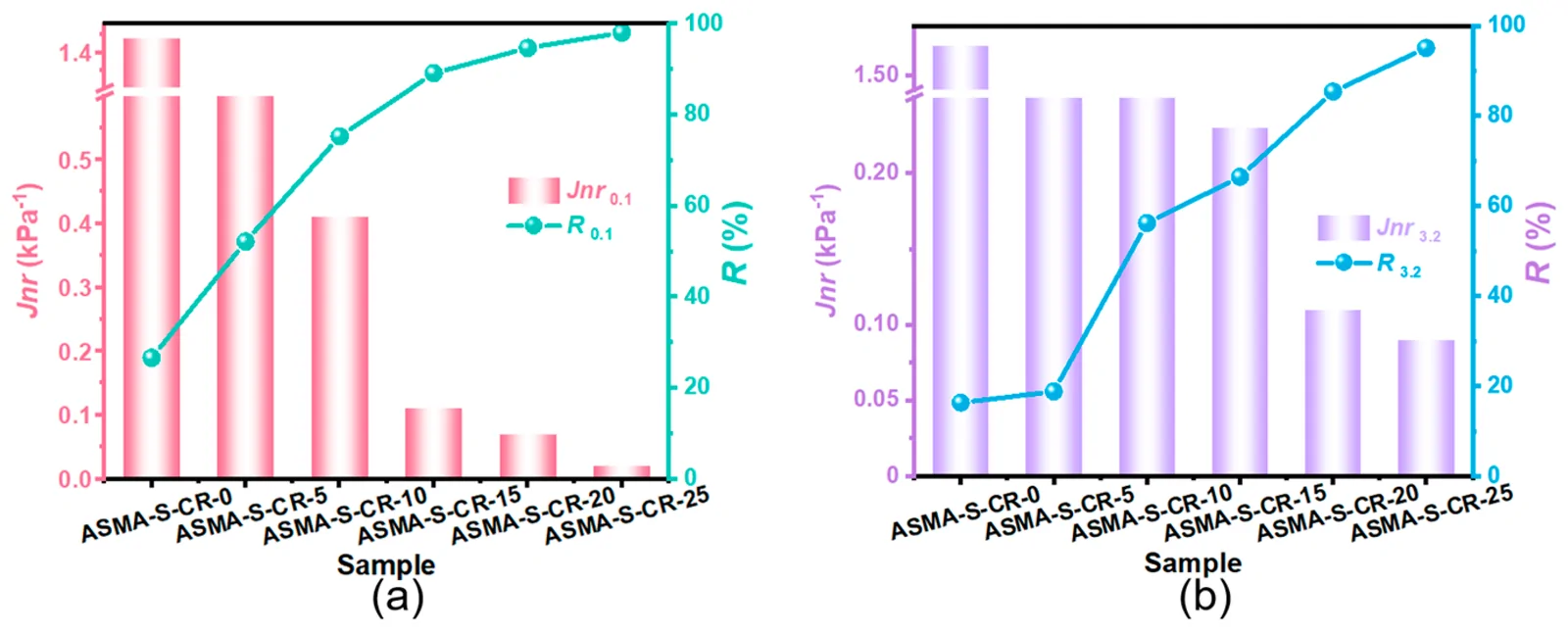
The (**a**) *Jnr* and (**b**) *R* of ASMA-S-CR-*x* at 64 °C.

The non-recoverable creep compliance (*Jnr*) represents the proportion of residual permanent deformation after stress loading, while the average creep recovery rate (*R*) reflects the elastic deformation recovery capability of asphalt after unloading. Both parameters are calculated from the multiple stress creep and recovery (MSCR) test and serve as key indicators for evaluating the high-temperature rutting resistance of asphalt [23]. As illustrated in Figure 5a,b, a consistent rheological feature can be observed. For all tested rejuvenated asphalts, the *Jnr* value at the high stress level of 3.2 kPa is higher than its counterpart at 0.1 kPa, while the *R* value at 3.2 kPa is significantly lower than that at 0.1 kPa. This phenomenon suggests that the internal viscoelastic network of rejuvenated asphalt is susceptible to structural damage under high loading stress, presenting measurable stress sensitivity. Across the full stress range, as the CR content gradually increases from 0 to 25%, the *Jnr* of all samples decreases monotonically in a gradient manner, while the *R* value rises synchronously and monotonically. This evolution law is consistent with the performance variation of G*/sin*δ* and G* described previously, supporting the rheological results. The above results indicate that the incorporation of CR can improve the permanent deformation resistance of rejuvenated asphalt, and the modification effect strengthens with increasing CR content. This performance improvement is mainly attributed to the physical filling effect of CR in the asphalt matrix. The dispersed CR particles fill weak regions inside the asphalt matrix and restrict the slippage and flow of asphalt molecular chains; their inherent elastic properties also contribute additional elastic recovery capacity to the system, thereby enhancing high-temperature rutting resistance. This conclusion is in good agreement with the existing published research in this field [29,30].

### 3.3. The Fatigue Properties of Rejuvenated Aged SBS-Modified Asphalt

**Figure 6:**
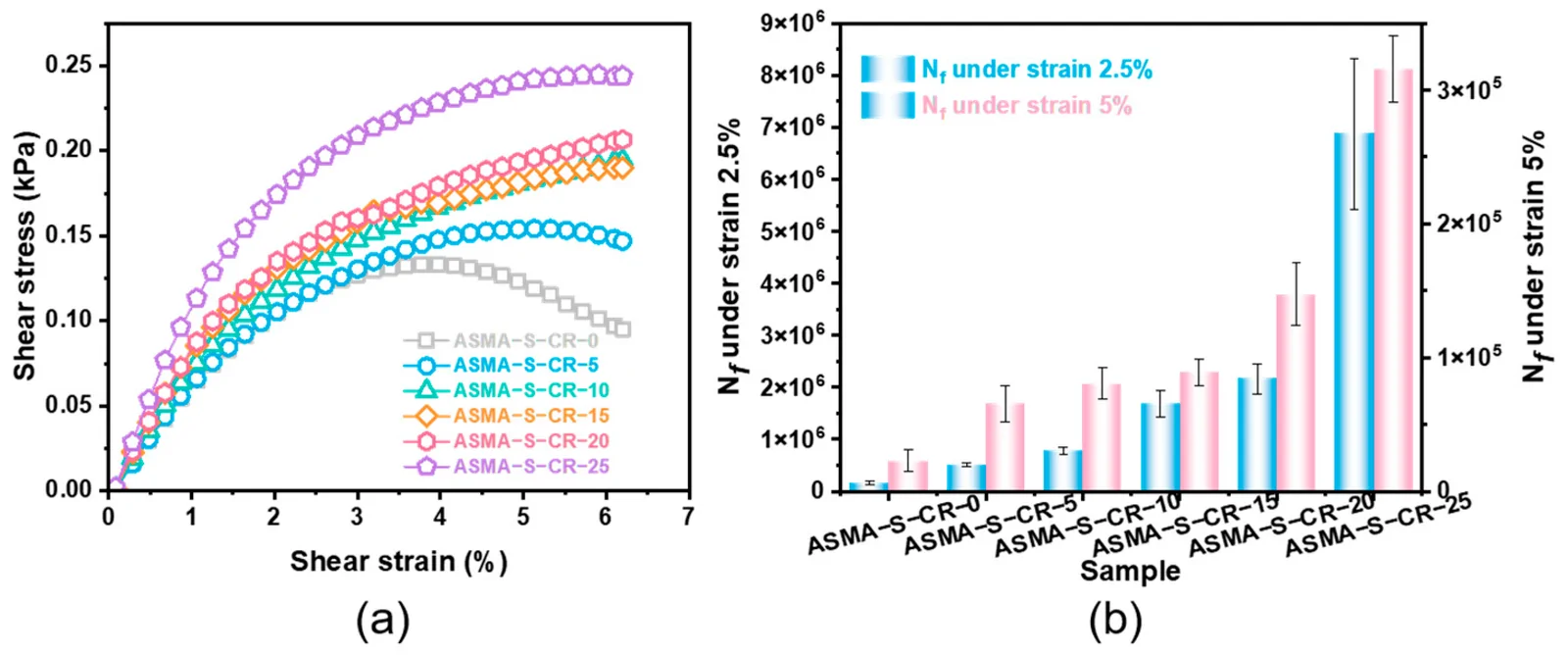
The (**a**) shear strain–stress curves of LAS test at 25 °C and (**b**) fatigue life of ASMA-S-CR-*x*.

The shear stress–shear strain curves obtained from the LAS test are presented in Figure 6a. The curves show that the shear stress of ASMA-S-CR-0 and ASMA-S-CR-5 (samples with low CR content) gradually rises to a maximum with increasing strain and then decreases to varying degrees. The staged evolution of shear stress during loading indicates that, at the initial loading stage, the gradual accumulation of internal microscopic fatigue damage corresponds to the continuous rise in the measured shear stress. After the stress reaches the peak value, the internal structure is damaged, and the rejuvenated asphalt cannot bear further deformation, manifested as a gradual decrease in stress in the curves [36]. The shear stress of the rejuvenated asphalt increases with increasing CR content, indicating that shear deformation resistance is enhanced as CR content increases. Furthermore, when the CR content increases to 10 wt%, no obvious stress peak or distinct strain softening phenomenon appears throughout the test, indicating that the rejuvenated asphalt still maintains a stable viscoelastic response at this temperature, without obvious fatigue failure. The statistical results of fatigue life under two typical strain levels of 2.5% and 5% are plotted in Figure 6b. The rejuvenated asphalt with low CR content shows relatively low fatigue life values at both strain levels, indicating relatively poor fatigue resistance. As the CR content increases, the fatigue life of the rejuvenated asphalt is several times higher than that of ASMA-S-CR-0, indicating a notable modification effect. At low CR contents, CR mainly acts as a dispersed phase in the asphalt matrix with limited improvement in fatigue life. After the continuous elastic network of CR is established, the system can dissipate strain energy more effectively under cyclic shear loading, thereby improving fatigue life.

### 3.4. The Thixotropy of Rejuvenated Aged SBS-Modified Asphalt

**Figure 7:**
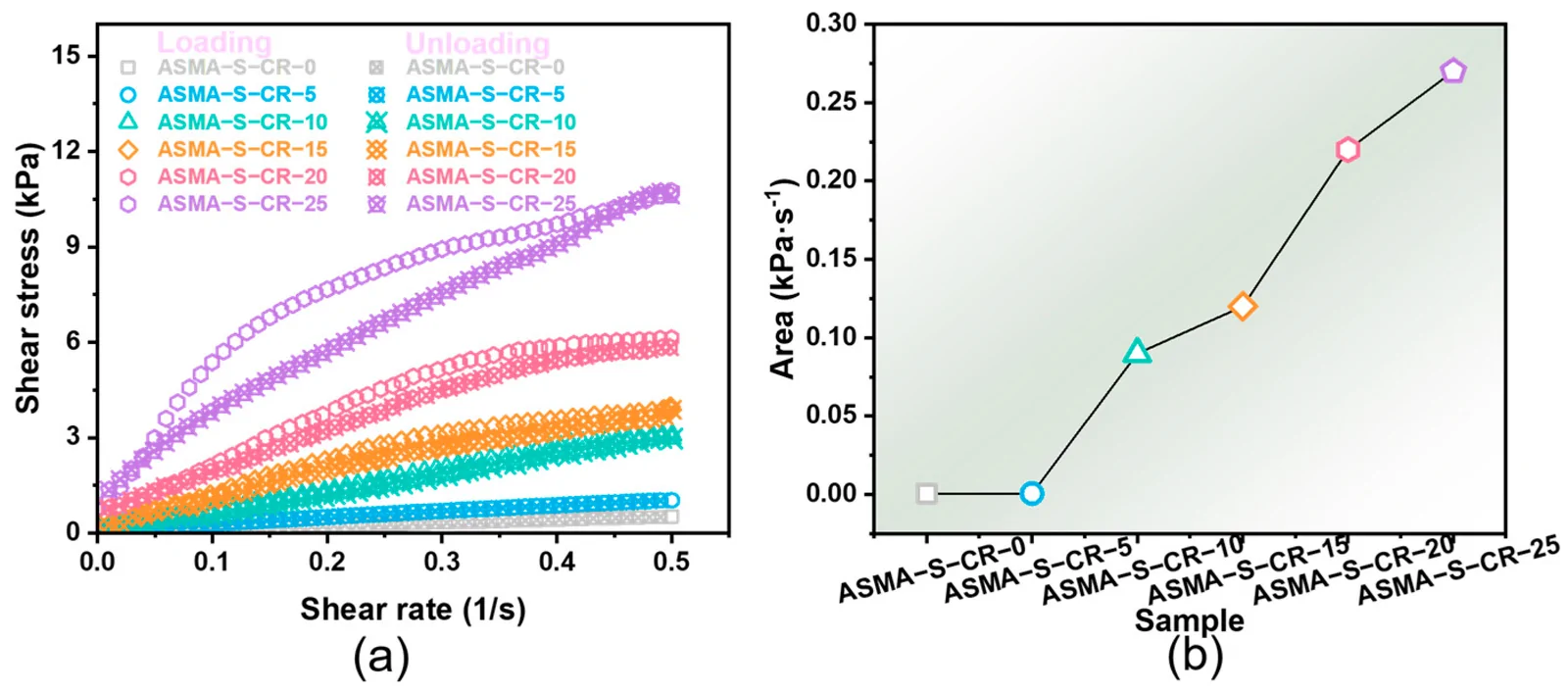
(**a**) The closed hysteresis loop of shear stress–shear rate curves and (**b**) the thixotropic loop area of ASMA-S-CR-*x* at 64 °C.

Thixotropy is one of the fundamental rheological properties of asphalt. The closed hysteresis loop of shear stress versus shear rate, namely, the thixotropic loop, can be obtained via DSR tests to quantitatively characterize the thixotropic behavior of asphalt [37]. The thixotropic loop area calculated by integrating the closed curve directly reflects the thixotropic strength of the system. The larger the loop area, the stronger the thixotropy, which indicates that the internal network structure of asphalt suffers a higher degree of destruction under shearing and that it takes a longer time to complete the structural self-recovery process [38]. The shear stress–shear rate thixotropic loops of different rejuvenated asphalts obtained at 64 °C are presented in Figure 7a. As the shear rate increases from 0 to 0.5 s^−1^, the shear stress of all samples increases monotonically, exhibiting typical shear-thinning rheological behavior. Across the entire shear rate range, the maximum shear stress of the samples increases with increasing CR content. The maximum stress of the CR-free rejuvenated asphalt (ASMA-S-CR-0) is less than 1 kPa, while the maximum stress of ASMA-S-CR-25 with 25 wt% CR content is close to 11 kPa, a difference of more than 10 times. For ASMA-S-CR-0, the loading and unloading curves almost overlap, indicating that there is almost no three-dimensional network structure that can be damaged by shear inside the rejuvenated asphalt without CR, and the rheological behavior is dominated by the viscous asphalt matrix, with little reversible structural destruction under shearing at high temperature. As the CR content gradually increases, the unloading curve shows a distinct downward shift, and the corresponding shear stress at the same shear rate decreases during the unloading stage. This phenomenon indicates that the elastic network constructed by CR inside the asphalt undergoes partial reversible structural destruction during shear loading, and the network structure gradually recovers with the corresponding stress reduction during unloading, which activates the thixotropy of the system. It is worth noting that when the shear rate returns to 0 during the unloading process, the stress of all samples returns close to the initial point, and the structural recovery is good within the scope of this test. This indicates that the internal network destruction of this series of rejuvenated asphalts with CR is a largely reversible process without irreversible structural damage, and the elastic network can largely recover to its initial state after unloading.

The calculated thixotropic loop areas of the rejuvenated asphalt binders are plotted in Figure 7b. It can be observed that ASMA-S-CR-0 or ASMA-S-CR-5 with only 5 wt% CR content barely exhibits thixotropy. As the CR content increases, the thixotropy of the binder increases. The increased thixotropic loop area of the high-CR-content system indicates that the composite network constructed by CR can undergo controllable structural destruction under shearing and provide a stronger driving force for elastic recovery after unloading, which is consistent with the improved permanent deformation resistance observed in the previous high-temperature rheological tests. The thixotropic loop area shows an approximately linear increase with increasing CR content, and the thixotropy is enhanced, which may provide a basis for inferring the phase transition of CR from a macroscopic rheological perspective. In the low-CR-content range, CR mainly acts as a dispersed phase in the asphalt matrix, and the system is still dominated by the viscous flow of asphalt, leading to a small increase in thixotropic loop area and limited improvement in thixotropy. As CR content further increases, CR particles gradually form a continuous phase, the dual-continuous network structure is established, and the proportion of elasticity in the system increases rapidly.

The phase transition of the composite materials is essentially a typical percolation phenomenon [39]. In this study, the classical power-law percolation model shown in Equation (1) was adopted to fit the thixotropy evolution data [40], so as to quantitatively calculate the critical content threshold for the connectivity transition of the CR phase. In this model, *P* refers to the measurable property, *n* is the power-law constant, *m* represents the CR content, and *m*_c_*P*’ is the percolation threshold. The fitted power-law equation is expressed as P=0.25×m−0.170.050.43. The coefficient of determination *R*^2^ of this fitting reaches 0.997, which demonstrates an extremely high fitting accuracy. Based on the quantitative calculation results derived from thixotropic performance data, the percolation threshold is determined to be 17 wt%, as a phase transition occurs within the system once the CR loading exceeds this critical value, whereby the CR phase transforms from an isolated dispersed state to a spatially connected continuous phase.

(1) $$P=\beta_{c}P^{\prime }{\left(\frac{m-m_{c}P^{\prime }}{m_{c}}\right)}^{n}\;\;m>m_{c}P^{\prime }$$

### 3.5. The Low-Temperature Rheological Properties of Rejuvenated Aged SBS-Modified Asphalt

**Figure 8:**
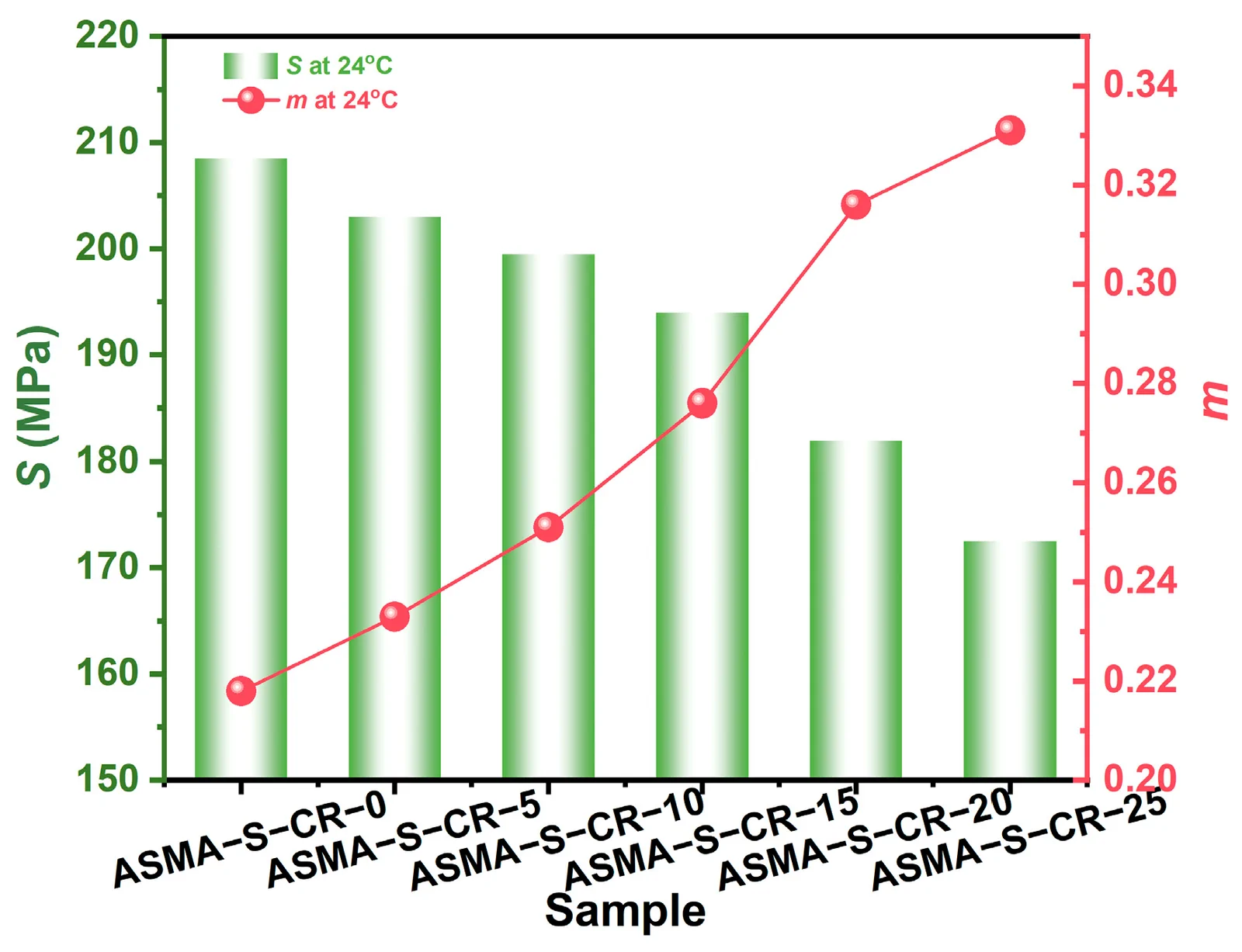
The stiffness (*S*) and creep relaxation rate (*m*) of ASMA-S-CR-*x* at −24 °C.

The creep stiffness (*S*) and creep relaxation rate (m) obtained from the BBR test are critical parameters for evaluating the low-temperature cracking resistance of asphalt. A smaller *S* value and a larger *m* value indicate better low-temperature deformation performance and stress relaxation capability [41]. In Figure 8, it can be clearly observed that the low-temperature performance is improved after CR addition, and the low-temperature cracking resistance improves with increasing CR content. This can be attributed to two complementary effects. On the one hand, the glass transition temperature of CR is far below the test temperature [42]; on the other hand, soybean oil penetrates into the interior of CR and acts as a plasticizer [43]. As a result, CR maintains an elastic rubbery state under the test conditions, reducing the low-temperature brittleness of the asphalt matrix. Combined with the high-temperature rheological performance, the secondary modification by incorporating CR on the basis of soybean oil rejuvenation not only enhances the high-temperature performance but also further improves the low-temperature performance of the rejuvenated asphalt.

### 3.6. The Micro-Structure Analysis of Rejuvenated Aged SBS-Modified Asphalt

**Figure 9:**
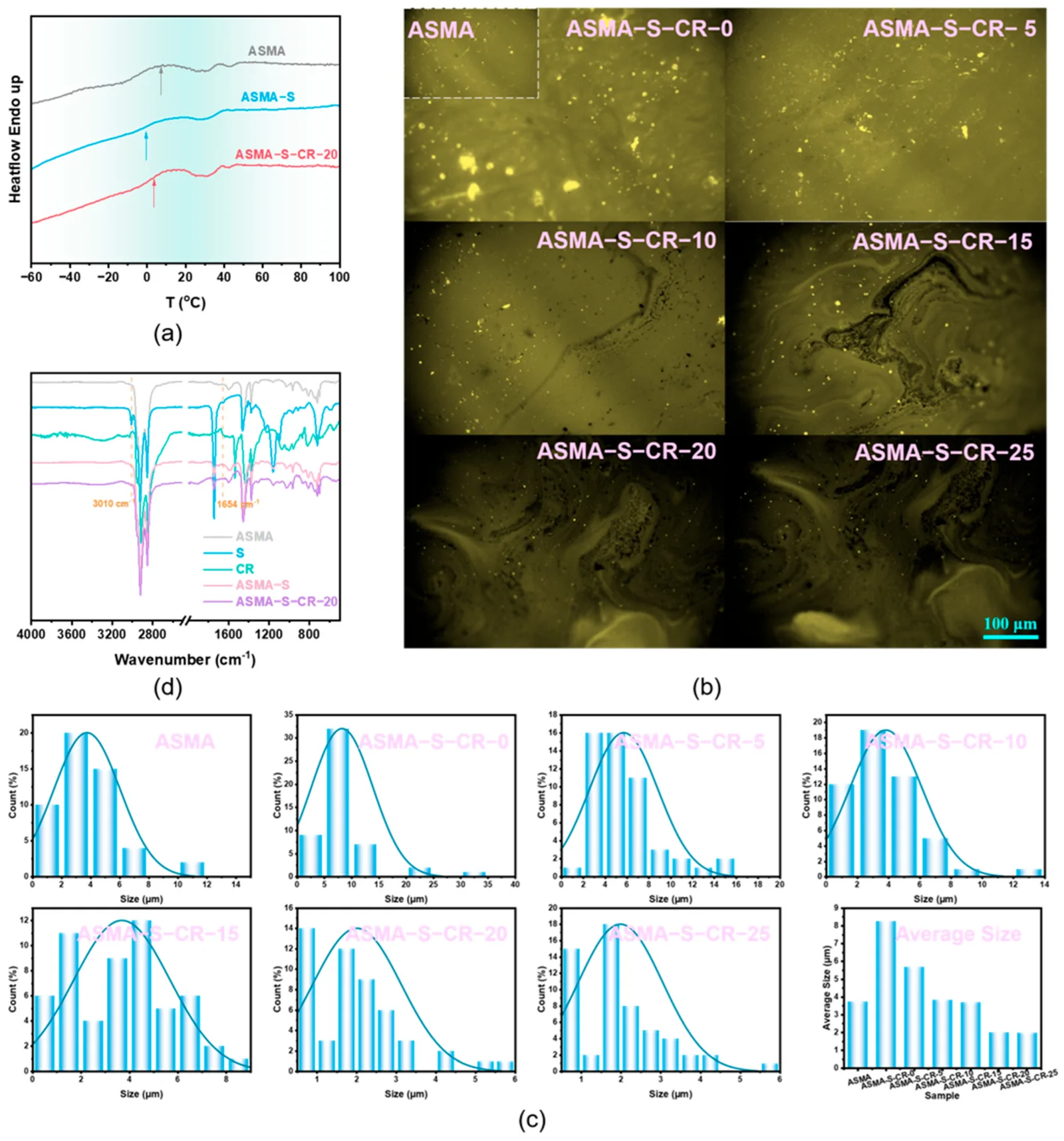
The (**a**) DSC curves and (**b**) fluorescence spectra of ASMA-S-CR-*x*, (**c**) size distribution curve of the residual SBS modifier and (**d**) FTIR spectra of different asphalts.

In Figure 9a, it can be seen that the glass transition temperature (*T*_g_) of ASMA is approximately 10 °C, which drops to around 0 °C after soybean oil rejuvenation (ASMA-S). This indicates that small-molecule soybean oil components diffuse into ASMA, weaken the intermolecular forces of heavy fractions in the aged binder, improve the segmental motion capability of asphalt molecular chains at low temperatures, and thus restore the low-temperature flexibility of the degraded asphalt system [44]. Subsequent addition of CR elevates the *T*_g_ of ASMA in ASMA-S-CR-20 back to 5 °C. This phenomenon is attributed to the fact that highly elastic CR particles construct a partially crosslinked elastic network in the asphalt matrix, and the interfacial interaction between CR and asphalt fractions restricts the free movement of partial light fractions in the aged asphalt, especially the soybean oil components. This helps prevent excessive deterioration of high-temperature rutting resistance while retaining the good low-temperature cracking resistance brought by soybean oil and achieves a synergistic balance between high-temperature and low-temperature rheological performance.

The fluorescence spectra of ASMA-S-CR-*x* are shown in Figure 9b. In these fluorescence microscopy images, the bright yellow domains correspond to the degraded residual SBS modifier, while the dark granular phases are dispersed CR particles. After ASMA is rejuvenated by soybean oil, the size of the residual SBS domains increases significantly. As shown in Figure 9c, the average particle size of the residual SBS modifier increased from 3.75 μm to 8.25 μm. The increase in SBS size indicates that soybean oil not only penetrates into the aged asphalt matrix but also induces a notable swelling effect on the residual SBS modifier and increases its particle size. With the subsequent incorporation of CR, the size of SBS domains decreased gradually. The higher the CR content, the smaller the SBS particle size, as the size of the SBS modifier in ASMA-S-CR-25 decreased to 1.99 μm. On the one hand, the size reduction of the residual SBS modifier can be attributed to the refinement effect under high-speed shearing; on the other hand, it can also corroborate the competitive adsorption of soybean oil between CR and the residual SBS modifier. The increased CR content synergistically forms a continuous co-existing phase with the residual SBS modifier in the asphalt system, which further improves the rheological stability of the modified asphalt binder [45].

The *T*_g_ results obtained from DSC testing provide solid thermal evidence for the confirmed synergistic rejuvenation effect between soybean oil and CR, indicating physical permeation behavior. In contrast, FTIR spectroscopic analysis can reveal molecular structure evolution, which helps clarify whether chemical interactions occur during the rejuvenation process. As observed in the FTIR spectra (Figure 9d), after ASMA is rejuvenated by soybean oil, the characteristic peak of C=C double bonds in soybean oil at 1654 cm^−1^ disappears, and the stretching vibration peak of C-H on the C=C double bonds at 3010 cm^−1^ nearly vanishes. This indicates that, unlike the purely physical rejuvenation effect of soybean oil widely reported in the existing literature [17,18], weak interfacial chemical reactions also occur during the rejuvenation process. This phenomenon is presumably attributed to the thermal-initiated cleavage of C=C double bonds, or the initiation effect of residual sulfur stabilizers in ASMA. Due to the highly complex composition of the asphalt binder and the limited sensitivity of the test instrument, the exact reaction pathway cannot be fully clarified at this stage. After incorporating CR, no obvious disappearance or generation of characteristic peaks is detected in the FTIR spectrum, which indicates that CR mainly plays a physical filling and swelling role in the modified asphalt system.

## 4. Conclusions

In this paper, a synergistic rejuvenation strategy combining soybean oil and CR was developed to address the long-standing performance trade-off of single-component bio-oil rejuvenated ASMA. The study explores the rheological evolution of soybean-oil-rejuvenated ASMA induced by CR content and elucidates the balance mechanism between the obtained high- and low-temperature properties. The following conclusions were drawn:

(1) Soybean oil rejuvenation effectively improves the low-temperature performance of ASMA but reduces its high-temperature performance. Based on comprehensive performance balancing, the dosage of 5 wt% is confirmed as a relatively reasonable choice for this system. At this dosage, the ductility reaches 36.2 cm, surpassing that of the original SMA, while the softening point is 62.0 °C. Once the soybean oil dosage is further increased, there is a notable decline in the softening point.

(2) Subsequent addition of CR further modulates the overall performance of the rejuvenated system. As CR content increases, the system gradually evolves from a dispersed phase filling state to a dual-continuous elastic network structure co-constructed by ASMA fractions and CR particles. This not only improves high-temperature rutting resistance and fatigue life under cyclic loading but also simultaneously reduces low-temperature creep stiffness and enhances the stress relaxation capacity, breaking the long-standing industry bottleneck that conventional rejuvenated asphalt cannot balance high- and low-temperature performance.

(3) Based on the characterization analysis of the microstructure, it can be inferred that soybean oil can not only physically permeate into the interior of ASMA but can also possibly utilize the active C=C double bonds on its molecular chain as initiation sites to trigger specific interfacial chemical reactions with the active components in ASMA. CR mainly exerts its modification effect through physical swelling, interfacial interaction and elastic network construction, without forming new chemical bonds in the system. The size of the residual SBS phase gradually shrinks as the content of CR increases. Combined with the *T*_g_ evolution pattern, it can be inferred that the possible competitive adsorption of soybean oil between CR and residual SBS modifier finally promotes the formation of a co-continuous phase of CR and residual SBS, which significantly improves the overall rheological stability of the rejuvenated binder.

The synergistic modification effect between a plant-based rejuvenator and solid waste CR is verified in this paper. However, the existing research results are currently limited to the asphalt matrix and the corresponding experimental conditions adopted in this work. Further exploration can be carried out in the following two aspects. On the one hand, process optimization can be conducted by referring to advanced technologies such as microwave-assisted desulfurization and surface modification in order to further reduce the construction viscosity of the high-concentration CR-modified system, improve the thermal storability, and solve the existing problem of high energy consumption in the wet process. On the other hand, the performance of asphalt mixtures can be verified and confirmed through systematic tests, including RAP blending efficiency, moisture absorption, rutting resistance, cracking resistance, water stability and other durability indicators, to promote this technology from the laboratory to large-scale engineering applications.

## Figures and Tables

**Table 1:** The conventional properties of SMA and aged SMA.

Sample	SMA	S-SMA	ASMA
Penetration (25 °C, 100 g, 5 s) (0.1 mm)	57.4	46.7	34.3
Softening Point (°C)	71.8	72.1	77.2
Ductility (5 cm/min, 5 °C) (cm)	31.0	14.8	0.4
Viscosity (135 °C), Pa·s	2.42	2.82	3.87

## Data Availability

The original contributions presented in this study are included in the article/Supplementary Material. Further inquiries can be directed to the corresponding author.

## Supplementary Materials

The following supporting information can be downloaded at https://www.mdpi.com/article/10.3390/ma19204274/s1, The captions for Supplementary Materials are listed below: Table S1. The basic characteristics of SBS modifier; Table S2. The basic characteristics of soybean-oil; Table S3. The basic characteristics of CR; Figure S1. The strain-sweep test of ASMA-S-CR-*x*.

## Abbreviations

The following abbreviations are used in this manuscript:

SMA	SBS-modified asphalt
RAP	Reclaimed asphalt pavement
AMSM	Aged SBS-modified asphalt/long-term aged SBS-modified asphalt
CR	Crumb rubber
DSR	Dynamic shear rheometer
BBR	Bending beam rheometer
S-ASMA	Short-term aged SBS-modified asphalt
ASMA-S	Soybean-oil-rejuvenated SBS-modified asphalt
ASMA-S-CR-*x*	Rejuvenated ASMA containing soybean oil and CR
MSCR	Multiple stress creep recovery
LAS	Linear amplitude sweep
ATR-FTIR	Attenuated total reflectance–Fourier transform infrared
DSC	Differential scanning calorimetry
*G**	Shear modulus
*G**/sin*δ*	Rutting factor
*Jnr*	Non-recoverable creep compliance
*R*	Creep recovery rate
*T_g_*	Glass transition temperature
