# Supplementary material for: Study on the Rheological Properties of Aged SBS-Modified Asphalt Rejuvenated with Soybean Oil and Crumb Rubber
Source: Materials (Basel). 2026 Oct 9;19(20):4274. doi: 10.3390/ma19204274 (PMC13706365; doi:10.3390/ma19204274)
Supplement: Supplementary file 1 [file materials-19-04274-s001.zip › materials-4577009-supplementary.pdf]

Supporting information for

Study on the Rheological Properties of Aged SBS-Modified Asphalt Rejuvenated with Soybean Oil and Crumb Rubber

Yang Chen [1], Chen Sun [1], Qinghao Ji [1], and Yujuan Zhang [1,*]

[1] College of Civil Engineering and Transportation, Yangzhou University, Yangzhou, Jiangsu Province, 225127, P. R. China; 008211@yzu.edu.cn

* Correspondence: 008211@yzu.edu.cn; Tel.: (+86-18762311612)

Table of Contents

The basic characteristics of SBS modifier……………..……………..……………..….S2

The basic characteristics of soybean-oil……………..……………..………………..….S3

The basic characteristics of CR………………………………………………… ………..S4

The strain-sweep test of ASMA-S-CR-*x*……………..………… ………..……………..S5

Table S1. The basic characteristics of SBS modifier

Properties	Value
Tensile strength (MPa)	10.3
Elongation at break (%)	836.0
Average molecular weight ($g{\cdot}mol^{-1}$)	290000
S/B ratio	30/70

Table S2. The basic characteristics of soybean-oil

Properties	Value
Viscosity at 60ºC ($mm^2 \cdot s^{-1}$)	22.3
Viscosity Ratio Before and After TFOT (%)	1.59
Mass Change Before and After TFOT (%)	-1.26
Relative Density ($g \cdot cm^{-3}$)	0.862

Table S3. The basic characteristics of CR

Properties	Value
Density ($g\cdot cm^{-3}$)	11.27
Ash (%)	5
Nature rubber (%)	65.44
Carbon black (%)	29
Rubber hydrocarbon (%)	68
Tensile strength (MPa)	3.89
Elongation at break (%)	177.00

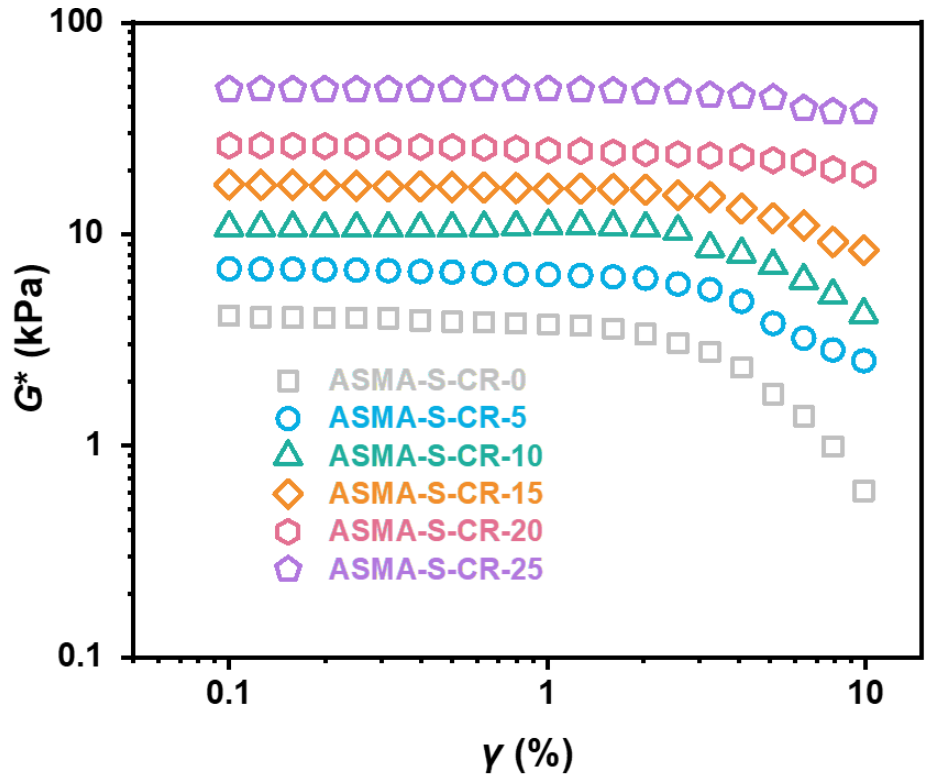

Figure S1 The strain-sweep test of ASMA-S-CR-*x*
